# Benefits of pentoxifylline for skin flap tissue repair in rats^1^

**DOI:** 10.1590/ACB351105

**Published:** 2020-12-18

**Authors:** Stephanie Luzia da Costa Pedretti, Cícero de Lima Rena, Laura Alejandra Ariza Orellano, Marcela Guimarães de Lazari, Paula Peixoto Campos, Tarcizo Afonso Nunes

**Affiliations:** I Fellow PhD degree, Postgraduate Program in Surgery and Ophthalmology, Department of Surgery, Medical School, Universidade Federal de Minas Gerais, Belo Horizonte – MG. Reproduction Biology Center, Universidade Federal de Juiz de Fora, Brazil. Substantive scientific and intellectual contributions to the study; conception and design; acquisition, analysis and interpretation of data; technical procedures; histopathological examinations; manuscript preparation and writing.; Reproduction Biology Center, Universidade Federal de Juiz de Fora, Brazil; II PhD, Department of Surgery, Medical School, Universidade Federal de Minas Gerais, Belo Horizonte-MG. Reproduction Biology Center, UFJF, Juiz de Fora – MG, Brazil. Substantive scientific and intellectual contributions to the study; conception and design; acquisition, analysis and interpretation of data; technicalprocedures; histopathological examinations; manuscript preparation and writing; critical revision.; Reproduction Biology Center, UFJF, Juiz de Fora – MG, Brazil; III PhD, Department of General Pathology, Institute of Biological Sciences, Universidade Federal de Minas Gerais, Belo Horizonte – MG, Brazil. Histopathological examinations, statistical analysis.; IV MD, Department of General Pathology, Institute of Biological Sciences, Universidade Federal de Minas Gerais, Belo Horizonte – MG, Brazil. Histopathological examinations, statistical analysis.; V PhD, Department of General Pathology, Institute of Biological Sciences, Universidade Federal de Minas Gerais, Belo Horizonte – MG, Brazil. Analysis and interpretation of data, histopathological examinations, statistical analysis.; VI PhD, Department of Surgery, Medical School, Universidade Federal de Minas Gerais, Belo Horizonte – MG, Brazil. Substantive scientific and intellectual contributions to the study; conception and design; acquisition, analysis and interpretation of data; technical procedures; critical revision; final approval.

**Keywords:** Pentoxifylline, Surgical Flaps, Wound Healing, Survival, Rats

## Abstract

**Purpose:**

To assess the action of pentoxifylline, administered by subcutaneous route, on skin flap tissue repair in rats, and to verify the histological aspects and biomarkers.

**Methods:**

Thirty-two male Wistar rats were divided into four groups: control (CT) and treated with pentoxifylline (P1, P3 and P5). Modified McFarlane technique flap was used. Ten days later, the animals were euthanized and the areas of viable and necrotic tissue were evaluated. Hematoxylin/eosin staining was used to assess the morphometric characteristics of the number of vessels and epithelial thickness. Picrosirius red was used to assess collagen density. VEGF and TGF-?1 levels on the skin flap and serum of the animals were measured by the ELISA method.

**Results:**

The macroscopic evaluation of the skin flap dimensions showed reduced necrotic tissue in the pentoxifylline (p < 0.05) treated groups. There was an increase in angiogenesis and reepithelization, demonstrated by analyses with an increased number of vessels (p < 0.05), VEGF and epithelial thickness. Fibrogenic effect showed decreased collagen density and TGF-β1 in the skin flap and serum.

**Conclusion:**

The benefits of pentoxifylline administered by subcutaneous route, at dose 100 mg/kg, which was effective to improve the survival of skin flap by acting on tissue repair components, stimulating angiogenesis and reepithelization, in addition to reducing fibrogenesis

## Introduction

The skin flap surgical procedure involves complex healing and circulatory processes, which start immediately upon incision, when the integrity of the skin is broken with vessel damage and consequent activation of the coagulation cascade pathways^1^,^2^. When making the flap, the vascular supply may be insufficient, especially in the distal segment, due to partial interruption of the vascular supply and consequent decrease in perfusion for a variable and transient period, which may compromise its survival and cause partial or total necrosis^3^-^5^.

The return of the oxygen supply to the ischemic tissue generates reactive oxygen species. Products resulting from the reperfusion mechanism influence and cause inflammatory and metabolic changes mediated by free radicals, which cause structural and functional cellular changes, which may contribute to tissue necrosis35,^7^.

The skin flap needs to survive and overcome all stages that lead to decreased perfusion and changes in the complex and sequential healing stages and, finally, achieve vitality throughout its extension, thus avoiding necrosis^1^–^4^.

The loss of vitality, especially in the distal segment, ranges from 9 to 65%, resulting in an impact on the patient’s quality of life, and is one of the reasons for increased health care costs, which must be avoided^1^,^3^–^5^. When present, flap necrosis is a challenge for the surgeon, as there will probably be a demand for secondary surgical procedures.

In several experimental studies^3^,^6^–^9^, drugs and other therapeutic modalities were used to mitigate the free-radical formation process, stimulate tissue repair and decrease tissue edema. Among these drugs, pentoxifylline has hemorheological properties^10^. The increase in blood supply, which causes more oxygen to be carried, allows the circulation of 3’,5’-cyclic adenosine monophosphate, enabling the metabolism to interfere with tissue vitality. Pentoxifylline acts on the coagulation cascade, inhibiting platelet aggregation and increasing prostacyclin synthesis – prostacyclin being an important vasodilator. It plays a role in morphological immunomodulation, decreases the inflammatory action, and has a fibrinogenic action, with decreased collagen synthesis. This potent antioxidant acts by inhibiting the superoxide anion synthesis and may reduce the toxic reperfusion metabolites, with a reduction in those responsible for tissue damage and consequent increase in flap survival^8^,^9^,^11^–^18^.

Pentoxifylline is administered by oral or parenteral route for circulatory disorders and occlusive peripheral arterial diseases^19^. In the literature, however, no publications were found on the use of pentoxifylline by subcutaneous route at a dose of 100 mg/kg. Thus, the purpose of this study is to verify the action of pentoxifylline on skin flap survival and tissue repair in rats at dose a local of 100 mg/kg.

## Methods

The experimental project was approved by the Animal Use Ethics Commissions (CEUA, Comissões de Ética no Uso de Animais) of the Universidade Federal de Minas Gerais (UFMG), Protocol No. 097/2016, and by the CEUA of the Universidade Federal de Juiz de Fora (UFJF), Protocol No. 036/2016. The ethical principles for animal experimentation were followed according to the guidelines provided by the National Council for the Control of Animal Experimentation (CONCEA, Conselho Nacional de Controle de Experimentação Animal), Brazilian Society of Laboratory Animal Science (SBCAL, Sociedade Brasileira de Ciência em Animais de Laboratório) and the Council for International Organizations of Medical Sciences (CIOMS). All protocols and procedures are in accordance with Act No. 11734, as of Oct 8 2008, and CONCEA-MCTIC^20^–^22^.

Surgical procedures were performed with laboratory animals at the Center for Reproduction Biology (CBR, Centro de Biologia da Reprodução) at UFJF. The samples analyses were conducted at the Apoptosis and Angiogenesis Laboratory of the Institute of Biological Sciences at UFMG, and the other financial costs were supplemented with personal resources.

The animals were kept in an air-conditioned shelf, under controlled lighting conditions (12-hour light/dark cycle), at an average temperature of 22 °C, 40–70% relative humidity, and *ad libitum* filtered water and animal feed (Nuvilab-Quimtia S.A., Colombo/PR, Brazil). Each animal was kept in an individual polypropylene 49 × 34 × 16-cm cage with a stainless-steel cover, and the floor was covered with wood shavings^2^,^20^–^22^.

### Animals

Thirty-two male Wistar rats (*Rattus norvegicus albinus,* Rodentia, Mammalia), with an average weight of 310 g and average age of 3 months, were randomly distributed into four groups of 8 animals, namely: control group (CT) and treated groups (P1, P3 and P5).

### Skin flap preparation

**Figure 1:**
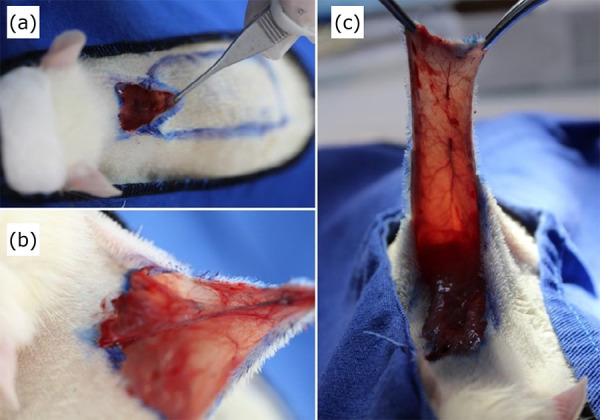
Skin flap preparation on the dorsum of a rat. **(a)** Drawing and early surgical skin flap incision on the dorsum of a rat; **(b)** and **(c)** Flap elevation.

The animals were intraperitoneally anesthetized with ketamine hydrochloride 10% at a dose of 90 mg/kg, and xylazine 2% at a dose of 10 mg/kg (ketamine hydrochloride 10%, Ketalex^R^, Vencofarma do Brasil Ltda Londrina/PR, Brazil; xylazine 2%, Sedalex^R^ Vencofarma do Brasil Ltda, Londrina/PR, Brazil). The surgical flap preparation technique was applied as proposed by Modified McFarlane *et al*.^23^ to the animals of the four experimental groups, as follows: antisepsis with degerming chlorhexidine digluconate 2% (Riohex, São José do Rio Preto/SP, Brazil); the dorsal trichotomy of the animal was performed with an area of approximately 10 × 4 cm (Home trichotomy machine, Carrefour Commerce and Industry Ltda)^21^. An incision on the marked area, a rectangular skin flap (2 × 8 cm), was made on the dorsum of the animal with a pedicle of the caudal vascular supply^23^,^24^; skin block detachment from the subcutaneous tissue up to the upper plane of the superficial muscle fascia; flap elevation and hemostasis under low pressure (Fig. 1). The flaps were sutured with a 4.0 nylon thread, with stitches 0.5 cm apart (mononylon, cuticular thread, Shalon^R^, Curvelo/MG, Brazil) in the four groups. The wounds were cleaned with a 100 mL bottle of 0.9% saline solution (Fresenius Kabi Unidade Aquirraz, Fortaleza/CE, Brazil) and gauze IV (Medhouse, São Paulo/SP, Brazil).

#### Use of medication on the flaps in the four groups of animals

**Figure 2:**
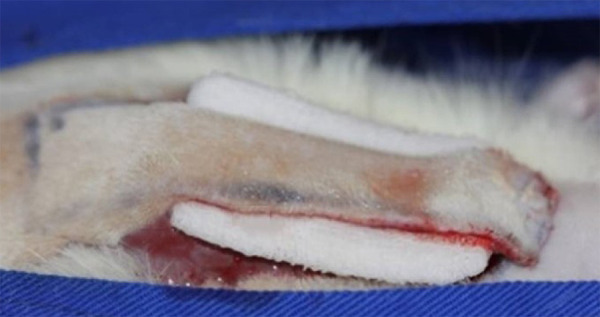
Group CT animal picture showing a rat dorsal flap with a saline solution 0.9%-soaked gauze above the muscle fascia, staying for 30 s.

**Figure 3:**
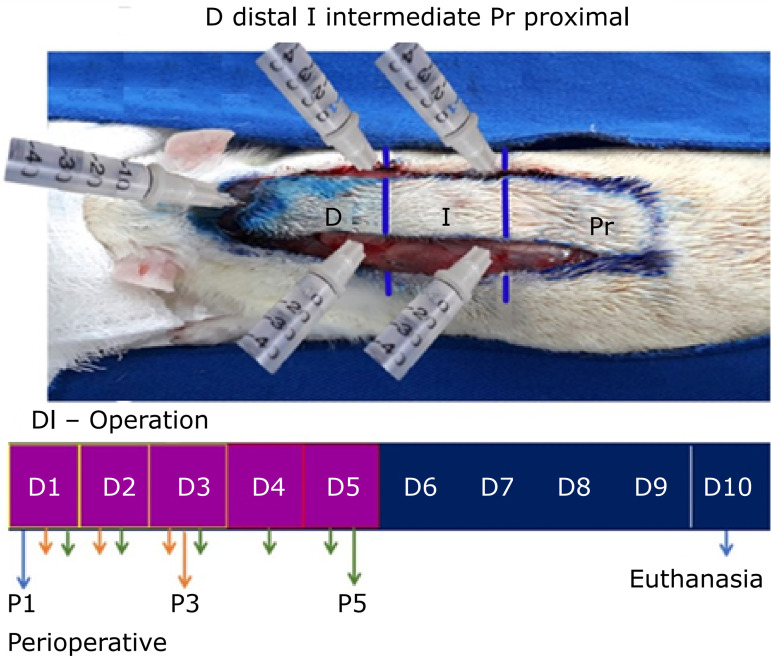
Schematic pentoxifylline administration in the treated groups (PI, P3, P5). Pentoxifylline was delivered at a dose of 100 mg/kg, adding twice as much distilled water to make 1 mL. The 1 mL content was divided into five 0.2 mL injections and delivered on the edge of the flap by subcutaneous route, as follows: top half of the distal segment; distal segment transition to the intermediate transition; intermediate to proximal; right side and left side. The flap was divided into 3 segments of 2.6 cm (total length approx. 8 cm).

The animals in the group CT (n = 8) underwent skin flap preparation and received a 0.9% saline solution-soaked gauze to moisten the flap bed for 30 s (Fig. 2). The treated groups (P1, P3 and P5, n = 8), received pentoxifylline (União Química, Pouso Alegre/MG, Brazil) at a dose of 100 mg/kg/day, which was added to bidistilled water to complete the 1 mL content (SaMtec, Ribeirao Preto/SP, Brazil). The 1 mL content of pentoxifylline solution was divided into five 0.2 mL injections to be applied on the superficial subcutaneous tissue at the edge of the flap, in the upper half of the distal segment, at the transition from the distal to the intermediate segment, and at the transition from the intermediate to the proximal segment on the right and left side. The 2 cm wide flap was marked in the distal segment to divide it in half (1 cm), and the 8 cm length was divided into three segments 2.6 cm apart (Fig. 3). The proximal segment was considered as the one closest to the vascular pedicle. The D1 of application of pentoxifylline was considered to be used in the subcutaneous flap in the perioperative period.

The animals in group P1 received 1 mL of the pentoxifylline solution by subcutaneous route in the perioperative period (D1) after the flap was prepared and before it was sutured. The animals in group P3 received 1 mL of the pentoxifylline solution on days D1, D2 and D3 in the perioperative period, 24 and 48 h after the operation, respectively. The animals in group P5 received 1 mL of the pentoxifylline solution on days D1, D2, D3, D4 and D5 in the perioperative period, 24, 48, 72 and 96 h after the operation, respectively (Fig. 3).

### Postoperative period

The animals operated on in the four groups (CT, P1, P3 and P5) received the analgesic pethidine – 10mg/kgby subcutaneous route, on the left side, 3.0 cm from the flap and tail, in the perioperative period (pethidine hydrochloride 50 mg/mL, Dolosal^R^, Cristália – Produtos Químicos Farmacêuticos Ltda. – Butantã-SP, Brazil). The prescription was with an administration interval of 8/8 h, for up to 3 days, as needed. In order to avoid overlapping doses, animals in groups P1, P3 and P5 received the pethidine analgesic (10 mg/kg) 30 min before pentoxifylline administration on days D1, D2, D3, D4 and D5, according to the animal group^21^. After receiving pethidine, the animals in groups P1, P3 and P5 were gently transferred to the container to receive pentoxifylline on the days that had been preset for these groups in the postoperative period (D1 for P1; D1, D2 and D3, for P3; D1, D2, D3, D4 and D5, for P5).

The animals were kept under observation in the recovery room and, when active, were taken to the temperature-controlled shelf of the Animal Experimentation Center (CEA, Centro de Experimentação Animal).

The macroscopic aspect of the skin flap of each animal was observed, and the tissue was considered viable if it had few or no changes in the superficial layer of the rat’s skin, i.e., similar to normal tissue. The tissue was considered necrotic if the rat’s skin had a hardened texture with a dark color ranging from brown to black, as well as crusts^26^.

### Flap exeresis, blood collection and euthanasia

The animals were euthanized by increasing the ketamine dose to 180 mg/kg (90 + 180 = 270 mg/kg) and the xylazine dose to 20 mg/kg (10 + 20 = 30 mg/kg) intraperitoneally.

Total flap, necrotic tissue and viable tissue area were measured, *in vivo*, with a digital caliper, and the macroscopic features of the flap on the tenth day (D10) after the operation were recorded and reported to the database for statistical analysis^9^,^26^. The photographic documentation occurred on days D1, D3, D5, D7 and D10, together with clinical observation of the flaps and all research animals by the same researcher during the experiment stages with a digital camera Cannon EF 100 mm, USM Tokyo, Japan.

Three animals from each group were randomly chosen to collect approximately 2 mL of blood (6 to 8% of the animal’s body weight^25^) by cardiac puncture to measure serum vascular endothelial growth factor (VEGF) and transforming growth factor beta 1 (TGF-ß1) levels. The blood collected was centrifuged (Excelsa II Centrifuge Model 206 BL-Fanem, São Paulo, Brazil, Harris Freezer – 80 ULT, Canada) to collect fresh plasma that was subsequently frozen in a freezer at -80 °C and taken for analysis at the ICB Laboratory at UFMG.

The rectangular surgical specimen of each animal was adhered to a filter paper mold, placed in a neutral (pH 7.4) 10% buffered formaldehyde solution vial for 48 h, labeled (10% buffered formaldehyde, 100 mL vial, Vetec PA, Rio de Janeiro/RJ)and sent for histological analysis. The vials containing serum and skin fragments from the proximal flap segment of the three randomly selected animals from each group were submitted for the biochemical measurement of VEGF and TGF-ß1.

### Histological and morphometric analysis

The sections were stained with hematoxylin-eosin (HE) and Picrosirius Red (PSR) for histological evaluation and morphometric quantification of the number of vessels, epithelial thickness, and collagen density.

Tissue repair phases in the flap segments were assessed at the transition from the viable tissue to the necrotic tissue on the skin flap. The histological evaluation and morphometric analyses were performed in the central portion of all slides in the three skin flap segments. Morphometry quantification of the number of vessels and the epithelial thickness for the proximal, intermediate, and distal segments were performed. Collagen density in the four groups was obtained from the capture and binarization of the images and densitometry of all histological section fields of the skin flap fragment^27^.

For the morphometric analysis of the histological elements, images of sequential sections of each skin segment were obtained: 25 fields (514.764 µm^2^) for the number of vessels, 10 fields (30.815 µm^2^) for epithelial thickness, and 20 fields (137.910 µm^2^) for collagen density^27^. The analysis, was made from digitized images obtained from the histological fields in a light microscope with a ×40 plan achromatic objective^9^,^14^,^27^.

The images were captured with a ×40 plan achromatic objective (final magnification = × 400), × 20 (final magnification = × 200) and × 100 (final magnification = × 1000), respectively; they were subsequently digitized with a JVC TK-1270/JCB microcamera and transferred to an analyzer (Image Pro-Plus 4.5 software, Media Cybernetics, Inc. Silver Spring, MD, USA) with the screen morphometry program Image Pro-Plus 4.5 with the thickness of the epithelium.

The PSR stain was used to quantify the collagen density. This stain is an anionic compound that differentiates the thickness and density of the collagen fibers based on the color that is emitted under polarized light. Light was used to view and identify the density of the collagen fibers based on the PSR staining in the center of each of the three flap segments (proximal, intermediate and distal). While the thin dissociated fibers are weakly birefringent and stained green, the denser, strongly associated fibers are intensely birefringent and emit longer-wavelength colors, such as red and yellow. The slides were analyzed with a ×10 objective. Images were obtained out of every 20 fields/slides in areas with a higher concentration of collagen fibers. Pixel densities for the regions occupied by the collagen fibers were selected on the real image for subsequent creation of a binary image for densitometric quantification^11^,^16^,^17^,^27^.

### VEGF and TGF-ß1 levels

The biochemical measurements of VEGF levels were used to quantify angiogenesis stimulation. The TGF-ß1 level was used to quantify the stimulation to collagen deposition^16^,^27^.

Eighty microliters of the remaining supernatant from the flap skin fragment sample were used to find VEGF and TGF-ß1 in the skin flaps and serum of the animals. Eighty microliters of the animal serum were used to measure the systemic levels. The VEGF and TGF-1 levels were measured in the skin flap sample (mean weight of 20 g) and in the blood serum of three animals (n = 3) from each group.

The enzyme assay followed the steps in the standardized protocol provided by the Kit Duoset (R&D Systems) supplier. Dilutions of cell-free supernatants were added in duplicate to the enzyme-linked immunoabsorbent assay (ELISA) plate, which had a rat-specific monoclonal antibody against the cytokine, followed by the addition of a second detection antibody. After washing to remove unbound antibodies, a substrate solution was added to the ELISA plate (50 µL of 1: 1 hydrogen peroxide solution and 10 mg/mL OPD). The reaction was stopped after 20 min of incubation with 2 N sulfuric acid (50 µL), and the color intensity was quantified at 540 nm in a microplate reader (Thermoplate). The standards were dilutions of recombinant rat cytokines from 7.5 pg to 1.000 pg/mL (100 µL). The results were expressed as cytokine picograms per milligrams of wet weight or milliliter of serum^27^.

### Statistical analysis and study variables

The sample calculation was based on enough investigated animals to achieve statistical representativeness for the analysis of the variables: total flap size, necrotic tissue size, viable tissue size, number of vessels, reepithelialization, and collagen density^2^,^20^,^22^,^25^. Data were analyzed with the contribution of the Biostatistics Center of the Medical School and ICB, both at UFMG.

All data were expressed as mean ± standard error of the mean (SEM). The assumptions of normality and homoscedasticity were determined by Kolmogorov–Smirnov tests for subsequent statistical analysis.

The level of significance was considered of 0.05, with a variation coefficient of 15 to 20% for the differences between the group CT and the pentoxifylline-treated groups P1, P3 and P5. The weight variable was evaluated to verify the animals’ health, as measured on the day of the operation (D1) and on the day of euthanasia (D10). The Newman–Keuls post-test was used to compare and detect significant differences between the averages in the four groups and variables with a parametric distribution, which are the measures of total flap size, viable tissue, necrotic tissue, collagen density, VEGF and TGF-ß1. The Kruskal–Wallis test and the Dunn’s post-test were used for nonparametric distribution data to compare and detect significant differences between the averages in the four groups.

The quantified tissue-repair variables were the number of vessels, epithelial thickness, and collagen density with eight animals per group. For the VEGF and TGF-ß1 measurements, there were three animals per group. The results were analyzed using the GraphPad Prism 5.0 software, and graphs were created based on the data in the tables.

## Results

All animals remained healthy during the experiment.

### Macroscopic analysis of skin flap survival

**Figure 4:**
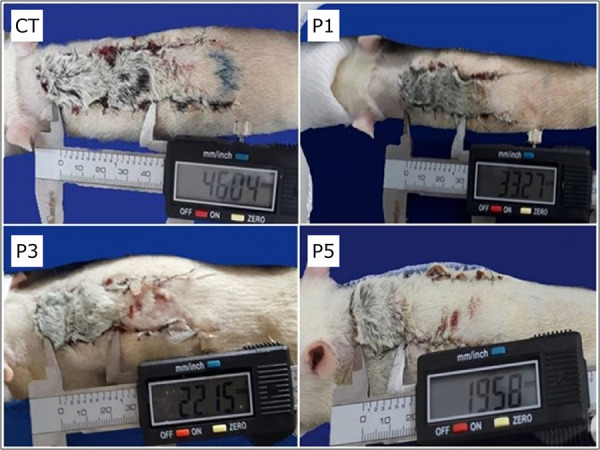
Representative measurements, *in vivo*, with a digital caliper showing the dimensions of tissue necrosis on the skin flaps. On Day 10 after surgery, compared to groups CT, P1, P3 and P5.

**Figure 5:**
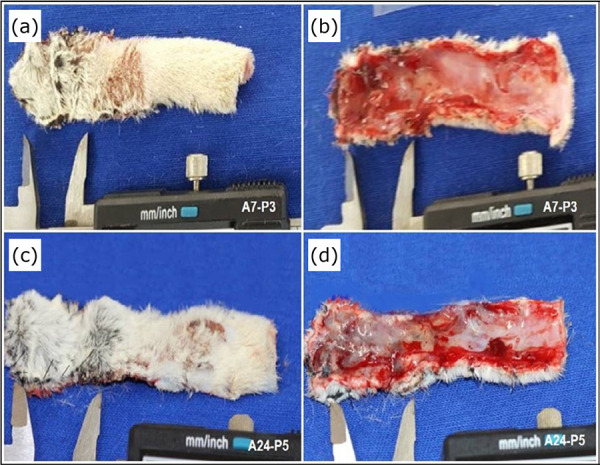
Rectangular dorsal and ventral skin surface of one of the animals in groups P3 **(a, b)** and P5 **(c, d)**.

Total flap area measured after 10 days of experiment were significantly reduced between CT and P5, p < 0.05. Significant differences were observed in the necrotic tissue area of CT compared with P3 and P5, showing that the necrotic tissue was reduced in treated groups P3 and P5 (Fig. 4). Viable tissue area in the treated groups was increased compared with the control group (Table 1). Treated groups showed necrotic tissue on the dorsal surface, and this necrosis was restricted to the superficial layer of the animal’s skin. Based on this correlation, it was observed that the ventral surface of these flaps had vital tissue in the layer below the necrosis (Fig. 5).

**Table 1:** Skin flap dimension: total, necrotic tissue and viable tissue (mm), mean, standard deviation (±) and percentage (%), comparison between CT, P1, P3 and P5; p-value.

Skin flap size	Mean (±) /% dimensions (mm)				p-value
	Group CT	Group P1	Group P3	Group P5	
Total	75.05 (± 1.38) 93.75%	69.62 (± 2.0) 87.03%	71.37 (± 0.90) 89.21%	68.58 (± 1.13)* 85.73%	0.0176
Tissue necrosis	35.85 (± 1.95) 43.75%	29.75 (± 2.12) 37.10%	23.,44 (± 1.55)*** 29.30%	22.73 (± 0.87)***^/0^ 28.41%	< 0.0001
Viable tissue	39.2 (± 1.62) 49.00%	42.45 (± 1.55)* 53.06%	45.74 (± 2.06)* 57.18%	45.85 (± 0.81)* 57.31%	0.0164

Significance:

* p < 0.05. Values are expressed as mean (±); n = 8 per group

*^/0^ Comparison groups: CT, P1, P3 and P5. Tests: ANOVA, Newman–Keuls post-test.

### Histological evaluation of skin flap samples

#### Morphometry of the number of vessels

**Figure 6:**
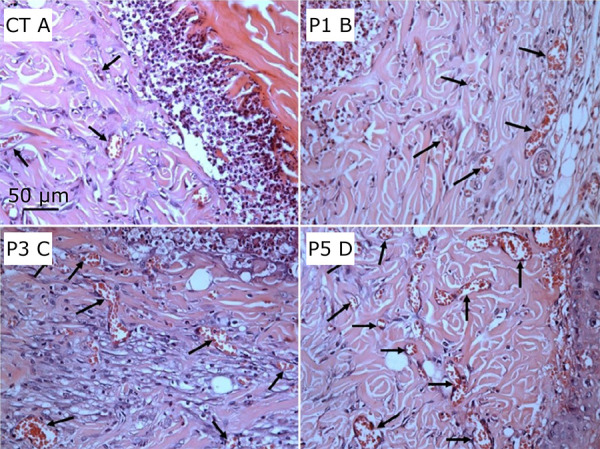
Photomicrographs of histological sections of the intermediate segment of granulation tissue samples in flap groups CT, P1, P3, P5. **(a – d)** Black arrows indicate vessels (HE staining, × 40 objective capture, × 400 magnification, 50 µm).

**Figure 7:**
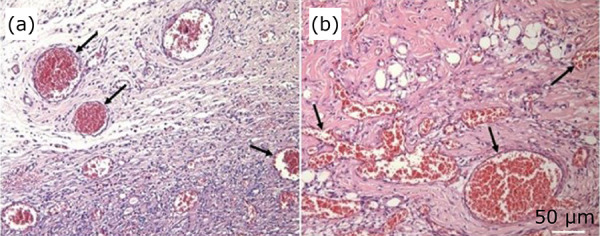
Photomicrographs of histological sections of the intermediate segment in the flap group P5 showing granulation tissue vessels.**(a, b)** Exuberant granulation tissue with numerous vessels – central intermediate segment (HE staining, ×20 objective capture, ×200 magnification, 50 µm).

When comparing the groups CT and P5, there was a significant difference in the proximal, intermediate, and distal segments of the skin flap, p < 0.05 (Table 2). The photomicrographs of the granulation tissue show increasing angiogenesis in the pentoxifylline-treated groups, this increase is more evident in P3 and P5 (Figs. 6 and 7).

**Table 2:** Morphometric analysis of the number of vessels/field (µm) for the proximal, intermediate, and distal segments for CT, P1, P3 and P5. Mean, standard deviation (±), comparison between CT, P1, P3 and P5; p-value.

Skin flap segments	Mean (±) number of vessels/field				p-value
	Group CT	Group P1	Group P3	Group P5	
Proximal	27 (± 0.8)	26 (± 0.2)	34 (± 0.2)***^/000^	41 (± 1.3)***^/000^	< 0.0001
Intermediate	34 (± 2.4)	34 (± 1.5)	36 (± 0.5)	50 (± 5.4)***^/000^	0.0007
Distal	21 (± 3.4)	23 (± 2.2)	30 (± 4.8)	40 (± 5.0)*^/0^	0.0174

Significance: *p < 0.05. Values are expressed as mean (±); n = 8 per group

*^/0^ Comparison groups: CT, P1, P3 and P5. Tests: ANOVA, Newman–Keuls post-test.

#### Epithelial thickness morphometry

**Figure 8:**
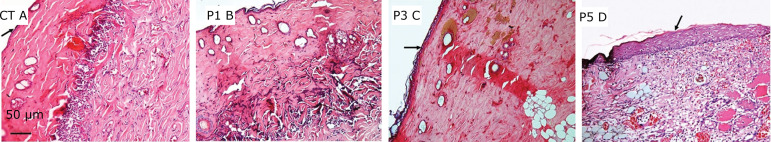
Photomicrographs of histological sections of the intermediate segment of granulation tissue samples in skin flap samples. **(a)** Subepithelial necrosis bordered by inflammatory infiltration and covered by a thin epithelial layer in group CT; **(b)** Evident subepithelial necrosis with increased inflammatory margin and a thin epithelium in group P1; **(c)** Inflammatory infiltrate decreasing and increasing epithelial thickness in group P3; **(d)** Slight inflammatory infiltrate, thicker epithelium with thinning of collagen fibers on the dermis, few areas of necrosis and vascularized granulation tissue; black arrows indicate increased epithelial thickness in group P5 (HE staining, ×20 objective capture, ×200 magnification, 50 µm).

Epithelial thickness increased in the three skin flap segments of the treated groups when compared with the control group(Table 3). A review of the intermediate segment samples, illustrated with photomicrographs, showed necrosis of the epidermis in all groups of animals, albeit most evident in the group CT. In the pentoxifylline-treated groups of animals, there was a continuous increase in epithelial thickness, p < 0.05 (Fig. 8).

**Table 3:** Morphometric analysis of epithelial thickness (µm) in proximal, intermediate, and distal flap segments in CT, P1, P3 and P5; mean, standard deviation (±), p-value.

Skin flap segments	Mean (±) epithelial thickness (µm)				p-value
	Group CT	Group P1	Group P3	Group P5	
Proximal	36 (± 3.4)	49 (± 3.5)	56 (± 5.8)*	58 (± 6.4)*	0.0116
Intermediate	13 (± 0.8)	16 (± 3.1)	23 (± 4.1)	28 (± 4.6)*	0.0146
Distal	9 (± 0.5)	10 (± 0.7)	11 (± 1.0)	15 (± 1.1)*^/000^	< 0.0001

Significance:

* p < 0.05. Values are expressed as mean (±). n = 8 per group

*^/0^ Comparison groups: CT, P1, P3 and P5. Tests: Kruskal–Wallis and Dunn’s post-test.

#### Collagen density morphometry

**Figure 9:**
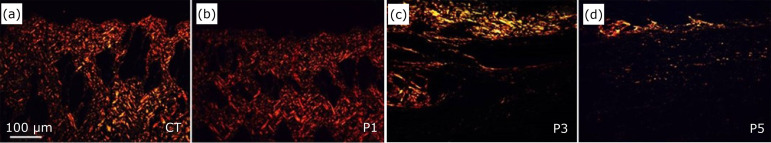
Photomicrographs of histological sections of the intermediate segment of skin flap samples from the groups. **(a – d)** A decrease in birefringence is seen in the groups, respectively; red and orange express strongly associated collagen fibers, birefringent in group CT (PSR staining, × 40 objective capture, × 400 magnification, 100 µm).

When comparing groups there was a decrease in collagen density in the three segments of the skin flap in the pentoxifylline-treated groups (Table 4). A higher collagen density was seen in the group CT with strongly birefringent red and orange fibers, which gradually decreased in the treated groups – group P5 being the lowest when compared to CT, p < 0.05 (Fig. 9).

**Table 4:** Morphometric analysis of collagen density (µm^2^) in the proximal, intermediate, and distal flap segments for CT, P1, P3 and P5. Mean, standard deviation (±), p-value.

Skin flap segments	Mean (±) collagen density (µm^2^)				p-value
	Group CT	Group P1	Group P3	Group P5	
Proximal	296.700 (± 19.680)	278.600 (± 25.310)	220.300 (± 15.000)*^/0^	184.600 (± 3.850)***^/00^	0.0014
Intermediate	177.100 (± 9.919)	137.900 (± 23.220)	106.100 (± 14.830)*	75.920 (± 1.110)***^/00^	0.0015
Distal	138.000 (± 3.330)	79.900 (± 11.620)**	60.270 (± 11.830)***	34.430 (± 5.321)***^/00^	< 0.0001

Significance:

* p < 0.05. Values are expressed as mean (±); n = 8 per group

*^/0^ Comparison groups: CT, P1, P3, P5. Tests: ANOVA, Newman–Keuls post-test.

### VEGF and TGF-ß1 biochemical measurements in the animal skin and serum

Vascular endothelial growth factor levels were higher in the skin compared with the serum of the animals, but it levels was increased in group P5 compared with P1 with a significant difference, p < 0.05 (Table 5). The TGF-ß1 levels were higher in the serum compared with the skin of the animals, but both showed continuously decreasing levels for this cytokine when comparing P1 with P5 with a significant difference, p < 0.05 (Table 6). The biomarkers were measured by the ELISA method.

**Table 5:** Vascular endothelial growth factor (VEGF) for CT, P1, P3 and P5, for the skin and serum. Mean, standard deviation (±), pg/mg, p-value.

VEGF level	Mean (±) VEGF (pg/mg)				p-value
	Group CT	Group P1	Group P3	Group P5	
Skin	246.8 (± 25.8)	333.6 (± 21.7)	274.6 (± 38.7)	471.1 (± 33.8)***^/00^	< 0.0001
Serum	145.2 (± 5.1)	153.9 (± 7.8)	132.9 (± 0.4)	223.9 (± 25.2)*^/0^	0.005

Significance: *p < 0.05. Values are expressed as mean (±). n = 3 per group

*^/0^ Comparison groups: CT, P1, P3 and P5. Tests: ANOVA, Newman–Keuls post-test.

**Table 6:** Transforming growth factor beta-1 (TGF-ß1) for CT, P1, P3 and P5, for the skin and serum. Mean, standard deviation (±), pg/mg, p-value.

TGF-ß1 level	Mean (±)TGF-ß1 (pg/mg)				p-value
	Group CT	Group P1	Group P3	Group P5	
Skin	162.1 (± 22.9)	93.8 (± 25.9)	73.0 (± 26.9)	46.4 (± 17.20)*	0.0187
Serum	313.9 (± 28.5)	259.1 (± 20.5)	225.8 (± 23.3)	191.3 (± 15.9)**	0.0063

Significance:

* p < 0.05. Values are expressed as mean (±). n = 3 per group

*^/0^ Comparison groups: CT, P1, P3, P5. Tests: ANOVA, Newman–Keuls post-test.

## Discussion

The animals were observed throughout the 10 days of the experiment to evaluate tissue repair, as the complete formation of the secondary vascular pedicle occurs around the 9th day^2^,^3^.

Pentoxifylline has a classical therapeutic indication for the treatment of circulatory disorders and occlusive peripheral arterial diseases, studies have administration included a protective effect against flap necrosis, when administered orally and parenterally. Even though, administration of pentoxifylline by subcutaneous route has minimal systemic repercussions, over the oral route, and it does not require repeated doses to reach the desired serum level^3^,^9^. And provides effective fluid absorption, adequately uniform and slow dispersion, concentration at the site where the action is expected to occur, and easy application^3^,^9^,^23^,^24^. The effects of pentoxifylline administered by subcutaneous route suggest the need to deepen knowledge to expand the domain.

Importantly, a significant improvement in tissue repair was observed in previous research, when 20 mg/kg of pentoxifylline and 5% dimethyl sulfoxide were administered by subcutaneous route in the perioperative period in the flap skin model^23^,^24^. Pentoxifylline in a 20 mg/kg dose was able to enhance the healing process on the skin flap with granulation histological elements was observed, such as exuberant neovascularization, inflammatory infiltrate with leukocytes in the pentoxifylline group, in addition to the absence of samples with necrosis in the group treated with pentoxifylline^9^. These results led to new research with the proposal to increase the dose of pentoxifylline to 100 mg/kgand the frequency of its administration, in order to increase the viability of the skin flap.

The safety of the pentoxifylline dose was based on the median lethal dose (LD50), which is 195 mg/kg after intraperitoneal administration and 1.385 mg/kg after oral administration in mice. The LD50 of the drug in rats is 230 mg/kg after intraperitoneal administration and 1.770 mg/kg body weight after oral administration^8^,^9^,^12^. In the present study, animals in the 100 mg/kg pentoxifylline-treated groups remained healthy with no signs of distress during the experiment.

In the present study, the macroscopic analysis of the skin flap vitality showed an increase in the viable tissue area with a consequently decrease in the necrosis area in the pentoxifylline-treated groups. It is important to note that granulation tissue was composed of an exuberant vascular component in the dermis and hypodermis under the necrotic area, thus characterizing necrosis of the epidermis only. The presence of granulation tissue in the layers underlying the necrotic tissue may be an important factor for secondary surgical procedures when necessary in medical practice.

Other^8^,^9^,^11^,^13^,^14^,^16^-^19^,^28^–^32^ have demonstrated that parenteral administration of pentoxifylline with different schemes and doses ranging from 1 to 50 mg/kg, was able to improve tissue vitality.

In this research, the histological evaluation of the skin flaps showed an increase in the number of vessels in the proximal, intermediate and distal segments of groups P3 and P5 compared with P1, i.e., in the groups with a long administration period of pentoxifylline during the postoperative period. Moreover, an increase in angiogenesis occurred in the three skin flap segments, and the effects of pentoxifylline were observed already with three days of pentoxifylline administration, including in the distal segment of the flap, which is the one furthest away from the vascular pedicle. An increase in the number of vessels was also observed under the flap necrotic area compared with group CT.

The proangiogenic effects of pentoxifylline on ischemic tissues have been observed in several studies. The administration of intradermal hyaluronidase combined with intraperitoneal pentoxifylline for 14 days showed a decrease in the cellularity of macrophages on the dermis, while the administration of pentoxifylline alone stimulated angiogenesis^17^. The biochemical and histopathological findings suggest that low doses of pentoxifylline can effectively improve ischemic damage in the experimental model of skin flaps^30^. The administration of 20 mg/kg/day of pentoxifylline by intraperitoneal route in rats undergoing myocutaneous flap of the caudal monopedicled rectus abdominis showed changes in the microcirculation system, decreased necrosis, and increased number of capillaries in the flap ischemia zone (zone IV)^31^.

Considering skin flap reepithelialization in this study, the pentoxifylline-treated groups of animals had an increase in epithelial thickness in the three segments of the skin flap analyzed, and in the proximal segment there was an increase after the third day of drug administration. The morphometric analyses of epithelial thickness in the four study groups showed an influence and action of pentoxifylline, such as in the migration of keratinocytes that stimulated reepithelization, to complete the repair of the flaps, as the drug was administered for a long period of time. The flap necrosis area also showed increased epithelial thickness with granulation tissue formation in the dermis to the hypodermis.

In this study, the morphometric analysis showed a decrease in collagen density and greater organization of the connective tissue in the proximal, intermediate, and distal segments of the skin flaps, especially when pentoxifylline was administered for a long period of time. Evidence of these alterations was found after the third day of administration on all flap segments, and from the first day on in the distal segment. The flap necrosis area also showed decreased collagen density in the granulation tissue from dermis to hypodermis, suggesting pentoxifylline had an antifibrogenic action.

Despite different methodologies, the literature has published researches^1^,^11^-^18^,^33^ showing that pentoxifylline contributed to healing. Many of the cellular events associated with the migration of keratinocytes are induced and regulated by individual growth factors or a combination of growth factors that locally stimulate molecules found close to the injury^1^,^17^,^33^. The properties of pentoxifylline act on the collagen organization and synthesis, decreasing the extracellular matrix and, consequently, decreasing the production of collagen, glycosaminoglycans, fibronectin, in addition to increasing the activity of collagenases, were also reported by several researchers^12^,^13^,^16^-^18^. An *in vitro* research^12^ on the action of pentoxifylline showed that dermal fibroblasts exposed to pentoxifylline produced twice the collagenase activity produced by fibroblasts without the action of this drug. A comparative study^13^ between pentoxifylline (oral and intralesional) and triamcinolone (intralesional) in keloids showed similar clinical results with both drugs, suggesting that pentoxifylline can be used in the treatment of keloid lesions^15^,^18^.

Measurements of growth factors VEGF and TGF-ß1 were performed in the proximal segment of the flap in three animals only in each group, since the scope of the research was to verify the action of pentoxifylline on the elements of tissue repair. The results of these measurements were aligned with the investigation on the actions of pentoxifylline on the skin flap tissue repair in rats.

The VEGF levels were higher in the skin flap than in the serum of the animals, and both showed a significant increase in this protein in the treated groups. This result suggests that pentoxifylline stimulated the production/liberation of this important angiogenic molecule and corroborate with the results in the literature^32^.

The measurement of TGF-ß1 levels showed a significant decrease in the level of this growth factor in the skin and in the serum of pentoxifylline-treated animals. This fact suggests that the drug acted by reducing the release of TGF-ß1at the local and systemic level and is more pronounced in the groups of animals receiving pentoxifylline, administered by subcutaneous route, for more days, suggesting an antifibrogenic role for the drug. The granulation tissue in groups P3 and P5 had exuberant vascularization and greater organization of the collagen fibers, when compared with groups CT and P1. Other studies^12^,^13^,^15^ reported that pentoxifylline acts by decreasing TGF-ß1 levels and collagen synthesis.

The morphometry of epithelial thickness, number of vessels, collagen density, VEGF and TGF-ß1 levels, as well as the morphological analyses of the flap segments showed that pentoxifylline acts on the angiogenesis, with benefits in the survival and tissue repair of the flaps and, consequently, decreased necrosis.

These findings may help to enhance flap survival with the pharmacological strategy of an available drug administered by subcutaneous route, in addition to benefits such as affordable cost, easy administration, high therapeutic index, effective results, treatment feasibility, known mechanism of action when administered via oral and parenteral routes, bioavailability and protective effects on necrosis. Pentoxifylline suggests that it is dose-dependent, with a possible variability in the different effects based on the dose, schedule, route, and administration period^9^,^11^,^14^,^28^–^32^.

However, these results showed that the proposed scheme at a subcutaneous dose of 100 mg/kg/day, administered by subcutaneous route, was effective in increasing flap vitality and survival. Angiogenesis was stimulated, with increased number of vessels, increased VEGF, increased epithelial thickness – phases that integrate tissue repair, with a consequent decrease in tissue necrosis. It showed an antifibrogenic effect with decreased collagen density and decreased skin flap TGF-ß1 in rats. The relevance of the benefits provided by pentoxifylline, administered by subcutaneous route, in skin flaps of rats allows the inference of its effectiveness in the survival, protective effect on necrosis, angiogenesis stimulation, and decreased fibrogenesis.

There are certainly several other ways to investigate in order to elucidate and expand the knowledge on pentoxifylline and its applicability. The exact mechanisms of action when it is administered by subcutaneous route require further research to clarify the dose. The contribution of this research was to corroborate that pentoxifylline at a dose of 100 mg/kg, when administered by subcutaneous route, is an adequate option for another route of administration of the drug, which so far had been used via parenteral and oral routes.

The purpose of this study was to evaluate the action of subcutaneous pentoxifylline on tissue repair and the benefits of the drug, such as increasing tissue survival and decreasing skin flap necrosis in rats – which is a proven fact. However, even in the presence of necrosis in the epidermis, granulation tissue was formed in the layers of the deep dermis and hypodermis. This fact is important for the successful skin coverage in subsequent procedures, as it will decrease hospitalization time, patient distress, and the financial costs in future, if. In view of the antifibrogenic effect of pentoxifylline, studies with the drug at a dose of 100 mg/kg by subcutaneous route with a diversified scheme may also be proposed.

## Conclusions

Pentoxifylline administered by subcutaneous route, at dose of 100 mg/kg, increased survival and tissue repair in rats treated for a three and five days. The benefits of pentoxifylline, were characterized by an evident increase in angiogenesis shown by increase in the number of vessels, corroborated by higher levels of VEGF in skin and serum, increased epithelial thickness with increased reepithelization in the skin flap. There was also an antifibrogenic effect with decreased collagen density, corroborated by diminished of TGF-ß1 levels in the skin and animal’s serum.
